# Potential of modern circulating cell-free DNA diagnostic tools for detection of specific tumour cells in clinical practice

**DOI:** 10.11613/BM.2020.030504

**Published:** 2020-08-05

**Authors:** Jernej Gašperšič, Alja Videtič Paska

**Affiliations:** Medical Centre for Molecular Biology, Institute of Biochemistry, Faculty of Medicine, University of Ljubljana, Ljubljana, Slovenia

**Keywords:** cfDNA, NGS, personalized medicine, liquid biopsy, ctDNA

## Abstract

Personalized medicine is a developing field of medicine that has gained in importance in recent decades. New diagnostic tests based on the analysis of circulating cell-free DNA (cfDNA) were developed as a tool of diagnosing different cancer types. By detecting the subpopulation of mutated DNA from cancer cells, it is possible to detect the presence of a specific tumour in early stages of the disease. Mutation analysis is performed by quantitative polymerase chain reaction (qPCR) or the next generation sequencing (NGS), however, cfDNA protocols need to be modified carefully in preanalytical, analytical, and postanalytical stages. To further improve treatment of cancer the Food and Drug Administration approved more than 20 companion diagnostic tests that combine cancer drugs with highly efficient genetic diagnostic tools. Tools detect mutations in the DNA originating from cancer cells directly through the subpopulation of cfDNA, the circular tumour DNA (ctDNA) analysis or with visualization of cells through intracellular DNA probes. A large number of ctDNA tests in clinical studies demonstrate the importance of new findings in the field of cancer diagnosis. We describe the innovations in personalized medicine: techniques for detecting ctDNA and genomic DNA (gDNA) mutations approved Food and Drug Administration companion genetic diagnostics, candidate genes for assembling the cancer NGS panels, and a brief mention of the multitude of cfDNA currently in clinical trials. Additionally, an overview of the development steps of the diagnostic tools will refresh and expand the knowledge of clinics and geneticists for research opportunities beyond the development phases.

## Introduction

Personalized medicine and the transition of clinically applicable research into practice have been rapidly evolving since the end of the last century. When the Human Genome Project was completed in 2003 with an almost fully sequenced human genome, it was expected that it will give an important push in the elucidation of human genetic diseases. Today we understand the functioning of human metabolism, genetics, and epigenetics much better, and research progress has led to the development of more precise genetic diagnostic tools (*1*, *2*). Various analytical methods for the detection of cancer using circulating cell-free DNA (cfDNA) obtained by liquid biopsy have undergone research phases and are now awaiting approval.

Currently, the cancer treatment is performed with radiotherapy and/or systemic treatment, such as chemotherapy, use of growth factors, or biological therapy (*3*, *4*). The cancer tissue is formed from tumorigenic cancer stem cells that differentiate into different cell types leading to highly heterogenic cancer tissue, while genetic cancer mosaicism is confirmed if several cell karyotypes coexist in organism (*5*, *6*). Using a suitable analytical tool, genes can be systematically searched for somatic or hereditary mutations (Supplementary table 1). Cancer associated genes mostly encode DNA repair proteins, tumour suppressors, and transcription factors (Supplementary tables 2-6).

To search for genetic mutations in cancer, tissue samples are routinely obtained with tissue biopsy. Modern tissue biopsy is a low-risk procedure, but cannot usually be performed in the early stages of cancer due to the small size of an often heterogeneous tumour mass. In this case, the liquid biopsy, a body fluid collection, represents a beneficial alternative. It allows easy sampling, which can be used for mutation analysis of somatic or tumour cells. Namely, in body fluids apoptotic and necrotic tissue cells release DNA (genomic and mitochondrial) and RNA (*5*, *7*-*13*), that is fragmented into circulating cell-free DNA/RNA (cfDNA/cfRNA). In the presence of tumours, the circulating tumour DNA (ctDNA) is released into the surrounding fluid, which in most cases reaches the blood. Due to the transport from tissue to blood, cfDNA is usually degraded into fragments of 100-280 base pairs (bp), or 280-450 bp and 450-700 bp (di- or tri-lengths of nucleosomal DNA) (*14*). Cell-free DNA can be detected in plasma and serum, cerebrospinal fluid, saliva, stool, urine, and other body fluids (*13*, *15*-*18*).

Circulating tumour DNA contains information on somatic, hereditary, and acquired mutations. It is an important marker found in body fluids that can be detected during tumour cell apoptosis and necrosis. Cell-free DNA biomarkers are suitable for the detection of early disease stages, relapse control, treatment success, and the development of chemical resistance (*19*).

Sample preparation and sequencing for cfDNA is almost identical to genomic DNA (gDNA) in the analytical and postanalytical stages, while in the preanalytical stage it requires a completely different set of sampling and processing methods. Cell free DNA is collected from blood plasma fraction, fragmentation step is not needed.

In the analytical stage, cfDNA analysis by quantitative polymerase chain reaction (qPCR) or next generation sequencing (NGS) is identical to gDNA. Problems can be attributed to DNA artifacts or usage of different internal controls or reference materials.

In the postanalytical stages, technical errors due to the application of different quantification algorithms or discrepancies in calculation, interpretation, and reporting of the results still remain a major problem.

The method for ctDNA analysis with increasingly growing preference is NGS, with which clinically relevant mutations in ctDNA samples from cancer patients have been successfully sequenced (*20*). Five hundred sixty eight mutations involved in non-small-cell lung cancer, gastrointestinal stromal tumour, colorectal carcinoma, and melanoma were searched for in DNA samples of 40 cancer patients (*20*). However, the introduction of new DNA technologies requires new genetic training of health care providers; new professions have to be introduced (*21*).

We have performed a review of modern genetic ctDNA diagnostics approaches for the detection of mutations in cancer associated genes. In order to give medical readers an overview of currently available clinical tests, we included approved cancer diagnostic and companion tests. Additionally, we prepared potential panels of cancer genes for future transition into clinical practice.

## Genetic ctDNA diagnostic tools

Cancer is usually manually detected with mammography, colonoscopy, biopsy, and flexible sigmoidoscopy X-ray and computed tomographic (CT) colonography and later graded with histopathological imagery, which is time consuming and tedious task that requires considerable effort, expertise and experience of pathologists (*22*, *23*). Diagnosis is difficult due too late stage symptoms (*24*, *25*). New techniques use more high-tech approaches based on antibody specific labelling and DNA sequencing. The high-risk patients can be constantly monitored measuring the serum markers often in combination with ultrasonography (*26*).

Development of evidence based diagnostic methods, used to evaluate the test and guide the diagnosis, need to go through four stages: i) formulation of clinical question from patient’s disease is, followed by, ii) search of relevant clinical articles, iii) evaluation of evidence for its validity and iv) usefulness is needed to implement the evolved disease diagnosis into clinical practice. To solve the disease diagnostic problem, a complete analytical process has to be implemented: all phases of preanalytics (DNA sampling, dissolution, clean up, preconcentration and separation, storage), analytics (DNA mutation detection), and postanalytics (data analysis and interpretation) have to be developed. Preanalytical stage, sample collection, handling, and processing is an important step, as improper handling may lead to false diagnosis, while analytical and postanalytical problems are method dependent.

If tissue biopsy is possible, *in situ* hybridization (ISH) technique enables visual processing of mutation carrying cells through chromophore (chromogenic *in situ* hybridization - CISH) or fluorophore (fluorescence *in situ* hybridization - FISH). *In situ* hybridization technique is a technique where a probe – labelled single-stranded DNA or RNA – selectively binds to a specific target site of the cellular DNA or RNA (*27*). Detection can be performed through chromogenic or fluorescent signal analysis. Chromogenic *in situ* hybridization is used to determine gene amplification, gene deletion, chromosome translocation, and chromosome number (*28*). Fluorescence *in situ* hybridization additionally offers a multiplex option; it is possible to detect multiple targets in a single sample (*29*). *In situ* hybridization methods have certain advantages compared to other methods (Table 1).

**Table 1:** Comparison of modern techniques used for detection of cancer mutations

	**Sanger sequencing**	**NGS**	**qPCR**	**FISH**	**CISH**
Tumour biopsy	gDNA	gDNA	gDNA	gDNA in fixated cells	gDNA in fixated cells
Liquid biopsy	cfDNA	cfDNA	cfDNA	/	/
Sequence information	partial sequence	sequence	partial sequence	point mutation	point mutation
Time of analysis	7 days	3 days	4h	4h	4h
Precision	nucleotide resolution	nucleotide resolution	mutation resolution	mutation resolution	mutation resolution
Possibility of simultaneous sample analysis	-	+	+	-	-
Possibility of simultaneous gene analysis	+	+	-	+ (a few)	-
Costs of a few sample analysis	high	high	low	medium	low
Costs of high throughput analysis	high	low	low	medium	medium
+ – possible. - – not possible. NGS – next generation sequencing. qPCR – quantitative polymerase chain reaction. FISH – fluorescence *in situ* hybridization. CISH – chromogenic *in situ* hybridization. gDNA – genomic DNA. cfDNA – circulating cell-free DNA.					

**Figure 1:**
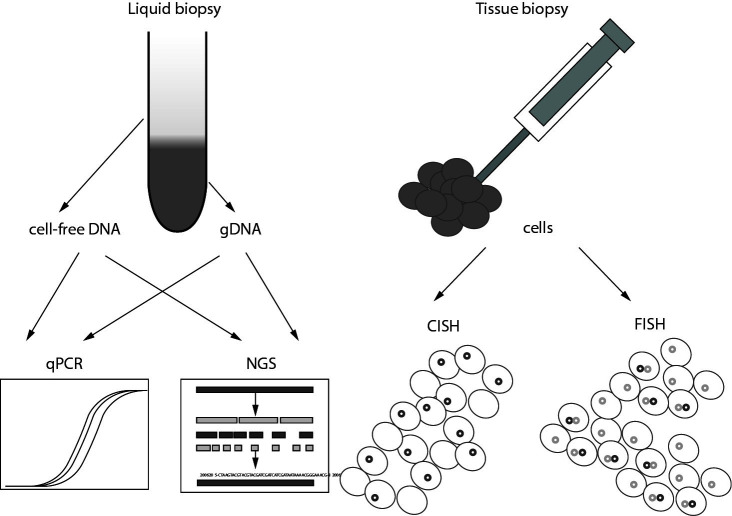
Scheme of sample collection and processing of data. Liquid biopsy - From blood isolation of circulating cell-free DNA and genomic DNA (gDNA) is possible. Genomic DNA has to be isolated from cells and represents mostly DNA from blood cells. Cell-free DNA is located in upper plasma fraction and contains DNA from apoptotic, necrotic cells. Tissue biopsy - To perform *in situ* hybridization analysis, cells have to be collected with tissue biopsy. CISH or FISH methods can be used to specifically detect target DNA or RNA mutation in tissue. After probe binding, samples can be observed under standard bright field microscope. CISH – Chromogenic *in situ* hybridization. FISH – fluorescence *in situ* Hybridization. qPCR – quantitative polymerase chain reaction. NGS – next generation sequencing.

Liquid biopsy enables easy sample collection, that can be used for mutation analysis of both somatic or tumour cells. Finger-stick capillary blood can be used as an alternative modern method for blood collection (*18*). Quantitative PCR or NGS enable fast, precise results. If the subpopulation of ctDNA is detected, tumour presence is confirmed. With the progression of the tumour, the share of ctDNA will increase (Figure 1).

## Quantitative and droplet digital PCR

A real-time polymerase chain reaction (real-time PCR), also referred to as qPCR, is a polymerase enzyme-based technique (*30*). The limit of validated qPCR methods is above 1% ct/cfDNA (*13*). Low detection is associated with method error; mostly it is attributed to technical error of preanalytical stage (standardization of sampling, sample storage, and preparation), analytical stage (use of different internal controls, reference material, for assessing the analytical performance, running analysis on different systems/platforms) or postanalytical stage (using different quantification algorithms, discrepancies in calculation and interpretation of the results, differences in reporting results) (*30*). The major problem (comparison of preanalytical, analytical, and postanalytical phases) of qPCR is that only a small number of genes can be analysed (Table 1). With an alternative method – the droplet digital PCR (ddPCR) we can determine ctDNA concentrations quantitatively and sensitively with better accuracy (*31*). The sample is sprayed into drops, where only one or zero copies of DNA exists (*32*). Droplet digital PCR then measures the signals in the absolute way as positive or negative (binary system). It is cheap, fast, but mutations must be tested sequentially; discovery of new mutations is impossible (*13*) (Table 1).

### Sanger sequencing

Sanger sequencing has been the main DNA sequencing technology for more than 30 years (*33*, *34*). This method is based on synthesizing DNA on a single strand DNA matrix, randomly integrating dideoxi-nucleotide chain terminators (*34*). In 1990, the method was upgraded to label terminators with different coloured dyes, so that all can be integrated into a single reaction (*35*). Sanger sequencing is perfect for DNA sequencing of tissue samples, while a small amount of ctDNA in liquid biopsy prevents sequential analysis of multiple target genes (*13*). Major problems of Sanger sequencing are high costs, low sample processibility, and long analysis time (Table 1). High background noise (associated with undesired priming, contamination, frame shift mutation, *etc.*) may lead to DNA sequence determination error.

### Next generation sequencing technology

Next generation sequencing methods are new technologies that are able to sequence a large number of samples with index-labelled DNA oligonucleotides (multiplexing). Next generation sequencing detectors monitor the addition of labelled nucleotides to already synthesized immobilized complementary DNA templates generated from the source DNA. Next generation sequencing systems offer reading lengths of 30-400 bp (*13*). Due to a large amount of information, the alignment of the sequences must be handled with the software. The software then automatically annotates the data with a variation/mutation database. The main NGS platforms are: Ilumina, Thermoscientific, BGI Genomics, Agilent Technologies, Qiagen, Macrogen, Pacific Biosciences California, Genewiz, 10x Genomics, Oxford Nanopore Technologies (*13*). In recent years, the affordability of NGS sequencing technology has lowered the price of whole genome sequencing (WGS). The costs for the WGS – entire genome sequence fell from 2.7 billion euros in 2003 to only 200 euros (on black Friday) and are even expected to fall (*36*).

From body fluid, blood cells or tissue, DNA can be isolated and further processed with special NGS preparation kits. DNA must be fragmented, repaired and adapter marked. There are several fragmentation methods that use ultrasound, enzymes, or chemicals. In the case of cfDNA, due to its fragmented state, no additional fragmentation procedure is needed.

The targets may be specific genes enriched with an NGS panel of cancer-related gene primers. The NGS panels can be custom-made or ordered (*e.g.* Ilumina, Agilent, LifeTechnologies). In this way, instead of WGS, sequencing is limited only to parts of the human chromosomes.

The NGS technique has several advantages over other methods (Table 1). It can be applied to all pathological conditions as it also enables the discovery of new DNA mutations. The major problem, the disadvantage of NGS is limited analytical ctDNA sensitivity (*13*), but the technology is evolving and sensitivity is expected to increase (Table 1). Low detection is associated with method error (incorrect calling of DNA bases or sequence variants), where artefacts in DNA sequence originate from preanalytical sample preparation, sequencing system platforms, or post sequencing data analysis. NGS enables the detection of somatic mutations under 5% (*37*). With the improvement of processes involved with sequencing, we can increase ctDNA detection in samples.

In clinical settings the NGS technology is already tested, but, is for now still too expensive to be used worldwide, due to high initial investment. The extra costs of specifically educated personnel and technology (material and service) are slowing down the possibility of global hospital use.

**Figure 2:**
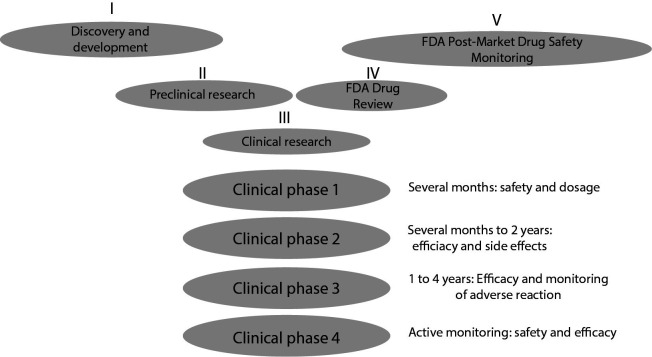
Phases of drug development (www.fda.gov). Development of drug is finished with preclinical *in vitro* and *in vivo* studies. Human drug effects are tested in clinical environment on patients with the condition/disease. Phases are divided into 4 phases: In phase 1 safety and dosage of the drug are determined on few subjects. In phase 2 efficacy and side effects are determined. If passed, drug goes into next phase that lasts from 1 to 4 years where efficacy and adverse reactions are monitored. In 4^th^ phase the drug is ready for the market, safety and efficacy are actively monitored. Food and Drug Administration (FDA) has to review drug documentation and later on monitor drug safety post-market.

All methods have to undergo rather long way to achieve standardization, pass through quality control in order to transfer into clinical practice (Figure 2).

## Cancer related candidate genes with potential of NGS panel assembly

The NGS cancer detection panel can be composed of a set of primers for genes involved in the specific tumour formation or tumour group. The sequencing of selected genes allows higher coverage and reduces analysis costs compared to whole genome sequencing. The advantage of the panel is that new genes can be easily added (*38*). Panels for breast, colon, hepatocellular pancreatic, and non-small cell lung cancer can be designed from the listed genes (Supplementary Table 2-6). Described cancers are spread worldwide and very difficult to detect at an early stage.

### Breast cancer

Breast cancer is the most common type of cancer in women worldwide (*39*). It is the second most common cancer. It is usually manually discovered on mammograms and later graded with histopathological images, which is a time-consuming and lengthy task that requires considerable effort, expertise, and experience from pathologists (*22*). New techniques use more high-tech approaches for cancer detection, such as antibody-specific labelling or sequencing of DNA. There is a connection between the degree of breast cancer and the mutation that caused it (*40*). However, enormous differences were found between inter- and intra-laboratory interpretations of the classification of breast cancer (*41*).

Data mining was performed in the scientific literature bases and internet sites: clinicaltrials.gov and the genetic testing registry database (*42*-*49*). Mutations in *BRCA1* and *BRCA2* are responsible for 2/3 of familial breast cancer (*50*). The rest mostly cover mutations in genes *ATM, CHECK2, PALB2, PTEN, TP53*. The *ERBB2* (*HER2*) gene is the most sequenced gene in recent diagnostics. Genes involved in breast cancer, suitable for the development of diagnostic tools are *TP53, CDH1, PALB2, ATM CHEK2, RAD51D, BARD1, BLM CDKN2A, FANCM MRE11A, RAD50, APC HOXB13* and *MITB* (*51*). Genes encode proteins that are involved in cell adhesion, cell growth, DNA repair mechanisms, and tumour suppression (Supplementary Table 2). Irreversible mutations in high-risk genes can cause damage that leads to the development of cancer cells and later somatic tumours (Supplementary Table 2). The database of clinical trials shows that several tests are waiting for approval. The genetic diagnostics were developed on the genes: *KRAS, PD-L1, ER, PIK3CA, BRCA1, BRCA2, BRCA2, EGFR, HER-2, C-MYC, PTEN, MET, IGFR-1*. Genes belong to the known oncogenes and tumour suppressors (clinicaltrials.gov). The Genetic Testing Registry NCBI database lists 34 genes used for breast cancer detection used in 613 tests: *AKT1, AR, ATM, BAPM, BARD1, BRCA1, BRCA2, BRCA3, BRIP1, CASP8, CDH1, CHEK2, CYP2D6, ERBB2, ESR1, HMMR, IL1B, IL1RN, KISS1R, KRAS, LFS3, MKRN3, NQ02, PALB2, PHB, PIK3CA, PPM1D, PTEN, RAD51, RAD54L, RB1CC1, SLC22A18, TP53, XRCC3* (*https://www.ncbi.nlm.nih.gov/gtr/*).

### Colorectal cancer

Colorectal carcinoma is one of the common wide-spread types of cancer (3^rd^ most common diagnosed malignancy, 4^th^ leading cause of cancer worldwide). It is usually treated with chemotherapy and EGFR antibodies (*23*). Early diagnostic methods for detection are colonoscopy, biopsy, and flexible sigmoidoscopy and computed tomographic (CT) colonography (www.nice.org.uk).

Gene candidates involved in cancer formation are stated in Supplementary Table 3 (*52*). Modern diagnostics for colorectal cancer detect mutations in *KRAS, NRAS,* and *EGFR* genes. Expressed proteins influence on cell proliferation and differentiation. High risk genes that can be used in the development of cancer diagnostics are *APC, MLH1, MSH2, MSH6, POLE, TGFBR2, MLH3, POLD1, MUTYH,* and *AXIN2* (*52*). Mostly proteins act as tumour suppressors or are involved in DNA repair (Supplementary Table 3) (*53*-*63*).

Clinical trials database holds information regarding mutations of *KRAS, NRAS,* and *BRAF.* In NCBI database genetic testing registry 33 genes are listed for colorectal cancer detection in 584 tests: *AKT1, APC, AXIN2, BUB1B, CRCS6, CRCS7, CTNNB1, DCC, DLC1, EP300, EPCAM; FGFR3, FLCN, GALNNT12, MLH1, MLH3, MSH2, MSH3, MSH6, MUTYH, NRAS, NTHL1, PIK3CA, PMS1, PMS2, POLS1, POLE, RNF43, SMAD7, SRC, TGFBR2, TP53, UGT1A1* (*https://www.ncbi.nlm.nih.gov/gtr/*).

### Hepatocellular carcinoma

Liver cancer is the sixth most common cancer. The most common type of liver cancer is hepatocellular carcinoma (*24*). High-risk patients are constantly monitored measuring the serum marker alpha-fetoprotein (AFP) often in combination with ultrasonography (*26*).

The tumour appears to be regulated by the Wnt/β-catenin signalling pathway. In hepatitis-induced hepatocellular carcinoma, β-catenin mutations are present in 13–41% of cases. In more than 55% of the cases, the mutations are present in the GSK-3β region of the β-catenin gene (*64*).

Diagnostics for hepatocellular carcinoma can be developed by screening high risk cancer genes for mutations in *CCNB1, CEP55, CHEK1, EZH2, KPNA2, LRRC1, PBK, RRM2, SLC7A11, SUCO, ZWINT* (Supplementary Table 4), that are up-regulated and *ACLS1, CDC37L1* (Supplementary Table 4), that are down-regulated (*65*). Up-regulated genes are involved in the process of duplication, differentiation, and the biosynthesis. Down-regulated genes are involved in biosynthesis of lipids and transcription of RNA (Supplementary Table 4) (*45*, *66*-*73*).

The vast number of hepatocellular carcinoma tests waits Food and Drug Administration (FDA) approval. Clinical trials tests screen whole genomes or specific genes for mutations (clinicaltrials.gov). In NCBI database ‘’genetic testing registry’’ 38 genes are listed for colorectal cancer detection in 94 tests: *ABCB11, APC, ATF7B, AXIN1, BMP2, CASP8, CCR5, CTNNB1, F5, FAH, G6PC, GPC3, GPC4, H19, HFE, HMBS, IFNAR2, IFN6, IFNGR1, IFNL3, IGF2, IGF2R, IL10RB, JAG1, JAK2, MET, MPV17, PDGFRL, PIK3CA, PTPRC, RSS, SERPINA1, SLC25A13, SLC37A4, SPRTN, TJP2, TP53, UROD,* (*https://www.ncbi.nlm.nih.gov/gtr/*).

### Non-small cell lung cancer

Lung cancer is the leading cause of death worldwide (*25*, *74*). It is graded as small cell and non-small cell lung cancer (NSCLC) types (*25*). NSCLC cancer is difficult to diagnose in early phases and first cancer signs are usually detected with X-ray and computed tomography (CT) (*25*).

Non-small cell lung cancer with mutations of epidermal growth factor receptor (*EGFR*) mutations, anaplastic lymphoma kinase (*ALK*) mutations, ROS proto-oncogene 1 (*ROS1*) rearrangement, mesenchymal-epithelial transition (*MET*) factor amplification, v-Raf murine sarcoma viral oncogene homolog B (*BRAF*) mutations, human epidermal growth factor receptor 2 (*HER2*) mutations, and *RET* rearrangement respond well to treatment (*75*).

Modern diagnostics for non-small cell lung cancer were developed on high risk cancer genes such as *EGFR, PD-L1, ALK, BRAF* (Supplementary Table 5), coding proteins involved in cell proliferation, and immune system evasion (Supplementary Table 5) (*76*-*88*).

More than 200 tests await in clinical trial settings. Genes *MET, KRAS, NRAS, EGFR, FGF, VEGF, PDGF, ALK, ROS1, HER2, HER3, BRAF* are tested in diagnostic kits for mutations. Genes belong to known oncogenes, responsible for proliferation, tumour suppression (Supplementary Table 5). In Supplementary Table 5 other candidate genes involved in NSCLC cancer formation are stated. In NCBI database ‘’genetic testing registry’’ 51 tests are listed for NSCLC cancer detection on genes: *ROS1, ALK, MET, ERBB2, KRAS, RET, EGFR, TYMS, RRM1, FGFR1, ERCC1, BRAF* (*https://www.ncbi.nlm.nih.gov/gtr/*).

### Pancreatic cancer

Pancreatic ductal adenocarcinoma (PDAC) is the most common type of pancreatic cancer. It is the twelfth most common cancer and the seventh most frequent cause of cancer-related death (*89*). Diagnosis is performed with CT or magnetic resonance (MR).

Familial pancreatic cancer mutations residues are located mostly on *BRCA1, BRCA2, p16, PALB2* genes. *BRCA2* mutations are highly associated with familial and sporadic pancreatic cancers (*90*).

Important genes determined to be involved in pancreatic cancer are stated in Supplementary Table 6 (*91*-*95*). Modern diagnostics for pancreatic cancer are still in the phase of clinical testing. Clinical research of diagnostics is being developed on high risk cancer genes such as *APC, ATM, BARD1, BRCA1, BRCA2, BRIP1, BMPR1A, CDH1, CDK4, CDKN2A, CHEK2, EPCAM, GREM1, MLH1, MRE11A, MSH2, MSH6, MUTYH, NBN, NF1, PALB2, PMS2, POLD1, POLE, PTEN, RAD50, RAD51C, RAD51D, SMAD4, SMARCA4, STK11, TP53* (clinicaltrials.gov). Researched proteins belong to the family of oncogenes, tumour suppressors. Some are involved in cell proliferation. In NCBI database genetic testing registry 28 genes are listed for pancreatic cancer detection in 287 tests: *AKT1, ATM, BARD1, BRCA1, BRCA2, BRIP1, CASP8, CDH1, CDKN2A, CHEK2, ESR1, HMMR, KRAS, NQO2, PALB2, PALLD, PHB, PIK3CA, PPM1D, RAD51* (https://www.ncbi.nlm.nih.gov/gtr/).

## FDA approved cancer diagnostic tests

The drug has to go through 4 clinical phases to determine its safety, efficiency, and dosage in advance of FDA approval (www.fda.gov). After initial discovery, a lot of time has to be invested into its development. Development is concluded with preclinical *in vitro* laboratory tests and *in vivo* animal studies. Safety is afterwards performed with tests on human subjects. Each of the clinical phases sections take a specific time period to complete the defined tasks (Figure 2). When clinical phases are completed, active monitoring of the drug begins. FDA reviews the drug documentation and in parallel monitor’s safety of the drug.

According to the FDA, companion diagnostic is a device that gives information regarding the safe and effective usage of the corresponding drug or biological product. Different genetic tests for mutation detection were developed and later approved by the FDA for different types of cancers (Supplementary Table 1). Mutation detection kits are gaining influence in clinical trials, where together with antibody and probe detection kits (immunohistological staining – IHS, western blot – WB) represents the majority of detection kits for use in personalized medicine (Supplementary Table 1).

Cobas *EGFR* Mutation Test v2 (Roche Molecular Systems, Inc.) was approved on 1 June 2016 (FDA) for the detection of non-small cell lung cancer. The test is part of a companion diagnostic with the cancer drug Tarceva (erlotinib) (*96*). It detects epidermal growth factor receptor gene mutations in non-small cell lung cancer patients (10-20% of all lung cancer) (FDA) (*96*). Cobas EGFR Mutation Test v2 was tested on blood samples of positive patients for 42 EGFR mutations on exon 18, 19, 20, 21 mutations as determined by the Test v1 (FDA). Drug Tarceva should work if one of the mutations is found in tumor DNA. Mutations can be discovered on gDNA and cfDNA samples.

Similar test the RealTime IDH1 Assay was approved in July 2018. A companion diagnostic test can be used to detect specific mutations in the *IDH1* gene in patients with acute myeloid leukemia (FDA). Tibsovo is an inhibitor of isocitrate dehydrogenase. The drug is administered if the test for gDNA mutation (isolated from white blood cells) comes out positive.

Approved diagnostic tests by FDA are optimized mostly for gDNA samples. Recently ctDNA technologies are gaining ground in the segment of companion diagnostics. In a few years, we expect a whole set of ctDNA diagnostics to be approved and enter clinical settings.

A large number of tests in clinical trials shows the importance of emerging technology. At the beginning of the 2020 year, 537 ctDNA and 368 cfDNA clinical test for different diseases were found to be awaiting approval (clinicaltrials.gov). Easy sampling (liquid biopsy) and processing needed for ctDNA testing is a major advantage of tests. The majority (non-ISH) of the already approved tests (Supplementary Table 1) can be modified to use cfDNA or ctDNA instead of gDNA, due to identical DNA source (cellular gDNA). In order to switch to cfDNA analysis, protocols for DNA isolation and DNA preparation for sequencing, need to be modified. Major points that should be addressed before handling the cfDNA are associated with the ability to isolate very small amounts of cfDNA and the prevention of leukocyte lysis. Therefore, during the transport extreme high and low temperatures, and agitations should be avoided. Plasma preparation using filtration or centrifugation should be performed prior to leukocyte lysis, within 6 hours after the blood draw using anticoagulant tubes K_2_EDTA. Furthermore, freeze-thaw cycles should be minimized to only one cycle in order to prevent nucleic acid degradation and the diminished capability of cfDNA detection. The isolation procedures vary, and numerous commercial kits are available. The versatility of purification protocols has an influence on the cfDNA purity and yield, which can reflect also on the downstream procedures, and therefore determination of optimal approach is crucial. In the sequential analytics step, it is of great importance to use appropriate reference material for the valuation of the analytical performance (*97*).

## Conclusions

This review describes the breakthroughs in modern diagnostic techniques that are recently approved or are in clinical trials. The overview will help geneticists to refresh the knowledge of drug or diagnostic development phases from the beginning and show which segment will surely prosper in the future.

Liquid biopsy analysis is becoming one of the important less invasive analysis tools. Precise knowledge of gene function and their role in the particular disease will help to detect causes of disease and prescribe preventive action. Genetic and epigenetic studies of oncogenes, tumour suppressors, and associated genes will shed new light on cancer development and diagnosis. Next generation sequencing developed analytical methods will bring a new era in precise personalized treatment, improve the usefulness and effectiveness of the medication.

The vast number of ctDNA and cfDNA tests registered in clinical phases shows the importance of new emerging technologies. FDA approved the first set of companion diagnostic tool in 1998 (*HER2*-trustuzumab). For cancer diagnostic whole set of companion diagnostic tools was approved later on. The tests use immunohistochemistry (IHC), *in situ* hybridization technique (CISH and FISH), PCR, qPCR, Sanger, and new NGS technology to detect specific mutation or overexpressed proteins (IHC). The diagnostic is coupled to the pharmaceutical drug that is very efficient for curing specific target tumours. Innovations in personalized medicine, such as precise genetic analysis of genome and acquired mutations, give new information regarding predisposition to certain diseases, and predict the effectiveness of the discovered drug. This allows a higher chance of recuperation.

In the future, the evolution of personalized medicine will enable the personalized treatment of disease/condition with the predicted course of treatment. The development of analytical tools will lead to the approval of urine cfDNA tests, which will even further facilitate sampling and screening of the patients. Most likely NGS panels for detection of cancers will be improved with a complete set of disease associated genes.

## Supplementary material

Supplementary Tables.
