# Supplementary material for: Potential of modern circulating cell-free DNA diagnostic tools for detection of specific tumour cells in clinical practice
Source: Biochem Med (Zagreb). 2020 Aug 5;30(3):030504. doi: 10.11613/BM.2020.030504 (PMC7394254; doi:10.11613/BM.2020.030504)
Supplement: Supplementary file 1 — Supplementary Tables. [file bm-30-3-030504-S1.pdf]

**Supplementary Table 1.** Table of FDA approved companion diagnostics (www.fda.gov). (Last accession date January 2020.)

| Type of disease | Approved tests | Diagnostic manufacturer | Analyzed genes | Mutations | Technique | DNA type |
|---|---|---|---|---|---|---|
| **Breast cancer** | BRACAnalysis CDx ™ | Myriad Genetic Laboratories, Inc. | *BRCA1* and *BRCA2* | Deleterious mutations | PCR, Sanger | gDNA |
| | FoundationOne CDx ™ | Foundation Medicine, Inc. | *ERBB2* (HER2) | *ERBB2* amplification | Genomic profile | gDNA |
| | INFORM HER-2/neu ™ | Ventana Medical Systems, Inc. | *HER-2/*neu antibody | / | Gene fluorescent probes (FISH) | / |
| | PathVysion HER-2 DNA Probe Kit ™ | Abbott Molecular Inc. | *HER-2* probe | / | Gene fluorescent probes (FISH) | / |
| | PATHWAY anti-Her2/neu (4B5) Rabbit Monoclonal Primary Antibody ™ | Ventana Medical Systems, Inc. | HER-2 antibody | / | IHC antibody | / |
| | InSite Her-2/neu KIT ™ | Biogenex Laboratories, Inc. | HER-2 antibody | / | IHC antibody | / |
| | SPOT-LIGHT HER2 CISH Kit ™ | Life Technologies Corporation | *HER-2* probe | / | Gene chromogenic probe (CISH) | / |
| | Bond Oracle HER2 IHC System ™ | Leica Biosystems | HER-2 antibody | / | IHC antibody | / |
| | HER2 CISH pharmDx Kit ™ | Dako Denmark A/S | *HER-2* probe | / | Gene chromogenic | / |

| | | | | | | |
|---|---|---|---|---|---|---|
| | | | | | probe (CISH) | |
| | INFORM HER2 Dual ISH DNA Probe Cocktail ™ | Ventana Medical Systems, Inc. | *HER-2* probe | / | Silver and chromogenic probe cocktail (CISH) | / |
| | HercepTest ™ | Dako Denmark A/S | *HER-2* probe | / | IHC antibody | / |
| | HER2 FISH pharmDx Kit ™ | Dako Denmark A/S | *HER-2* probe | / | Gene fluorescent probe (FISH) | / |
| | Therascreen PIK3CA RGQ PCR Kit | QIAGEN | *PIK3CA* | 11 mutations: exon 7: *C420R*; exon 9: *E542K*, *E545A*, *E545D* (1635G > T only), *E545G, E545K, Q546E, Q546R*; and exon 20: *H1047L, H1047R, H1047Y*: | qPCR | gDNA or ctDNA |
| **Ovarian cancer** | BRACAnalysis CDx ™ | Myriad Genetic Laboratories, Inc. | *BRCA1* and *BRCA2* | *BRCA1, BRCA2* deletions | PCR, Sanger | gDNA |
| | FoundationOne CDx ™ | Foundation Medicine, Inc. | *BRCA1/2* | *BRCA1/2* alterations | Genomic profile | gDNA |
| | FoundationFocus CDxBRCA Assay ™ | Foundation Medicine, Inc. | *BRCA1* and *BRCA2* | *BRCA1/2* alterations | NGS | gDNA |
| | Myriad myChoice® CDx | Myriad Genetic Laboratories | *BRCA1* and *BRCA2* | *BRCA1* and *BRCA2* SNV, insertions, deletions, rearrangements | NGS | gDNA |

| | | | | | | |
|---|---|---|---|---|---|---|
| **Non-small cell lung cancer** | Therascreen EGFR RGQ PCR Kit ™ | Qiagen Manchester, Ltd. | *EGFR* | *EGFR* exon 19 deletions, L858R | PCR | gDNA |
| | Cobas EGFR Mutation Test v2 ™ | Roche Molecular Systems, Inc. | *EGFR* | *EGFR* p.G719X, exon 18 mutations, exon 19 deletions, p.T790M, p.S768I, exon 20 insertions, p.L858R, p.L861Q | qPCR | gDNA or cfDNA |
| | PD-L1 IHC 22C3 pharmDx ™ | Dako North America, Inc. | PD-L1 antibody | / | IHC antibody | / |
| | PD-L1 (SP142) ™ | Ventana Medical Systems, Inc. | PD-L1 antibody | / | IHC antibody | / |
| | FoundationOne CDx ™ | Foundation Medicine, Inc. | *EGFR, ALK, BRAF* | *EGFR* deletions, p.L858R, p.T790M, *ALK* rearrangements, *BRAF* p.V600E | Genomic profile | gDNA |
| | VENTANA ALK (D5F3) CDx Assay ™ | Ventana Medical Systems, Inc. | ALK antibody | / | IHC antibody | / |
| | Oncomine Solid Tumor kits (Europe only) ™ | Life Technologies Corporation | *EGFR, ALK, ERBB2, ERBB4, FGFR1, FGFR2, FGFR3, MET, DDR2, KRAS, PIK3CA, BRAF, AKT1, PTEN, NRAS, MAP2K1, STK11, NOTCH1,* | all deletions, insertions, inversions, and substitutions in selected genes | NGS | gDNA, RNA (Fusion Transcript Kit) |

| | | | | | | |
|---|---|---|---|---|---|---|
| | | | *CTNNB1, SMAD4, FBXW7, TP53* | | | |
| | Vysis ALK Break Apart FISH Probe Kit ™ | Abbott Molecular Inc. | *ALK* rearrangement probe | / | Gene fluorescent probe (FISH) | / |
| **Acute myeloid leukemia** | Abbott RealTime IDH1 ™ | Abbott Molecular, Inc. | *IDH-1* | p.R132C, p.R132H, p.R132G, p.R132S, and p.R132L | PCR | gDNA |
| | Abbott RealTime IDH2 ™ | Abbott Molecular, Inc. | *IDH-2* | p.R140Q, p.R140L, p.R140G, p.R140W, p.R172K, p.R172M, p.R172G, p.R172S, and p.R172W | PCR | gDNA |
| **Chronic myeloid leukemia** | MRDx BCR-ABL Test ™ | MolecularMD Corporation | *BCR-ABL* rearrangement panel | *BCR-ABL* rearrangements | qPCR | RNA |
| **Melanoma** | FoundationOne CDx ™ | Foundation Medicine, Inc. | *BRAF* | *BRAF* p.V600E, p.V600K | Genomic profile | gDNA |
| | THXID BRAF Kit ™ | bioMérieux Inc. | *BRAF* | *BRAF* p.V600E, p.V600K | qPCR | gDNA |
| | cobas 4800 BRAF V600 Mutation Test ™ | Roche Molecular Systems, Inc. | *BRAF* | *BRAF* p.V600E | PCR | gDNA |
| **Colorectal cancer** | FoundationOne CDx ™ | Foundation Medicine, Inc. | *KRAS, NRAS* | *KRAS* wt, *NRAS* wt (Absence of mutations) | Genomic profile | gDNA |
| | Praxis Extended RAS Panel ™ | Illumina, Inc. | *KRAS, NRAS* | 56 *KRAS/NRAS* mutations | NGS | gDNA |
| | Cobas KRAS Mutation Test ™ | Roche Molecular Systems, Inc. | *KRAS* | mutations in codons 12, 13 and 61 of *KRAS* gene | PCR | gDNA |

| | | | | | | |
|---|---|---|---|---|---|---|
| | Therascreen KRAS RGQ PCR Kit ™ | Qiagen Manchester, Ltd. | *KRAS* | *KRAS* codons 12,13 | qPCR | gDNA |
| | Dako EGFR pharmDx Kit ™ | Dako North America, Inc. | EGFR antibody | / | IHC antibody | / |
| **Acute myelogenous leukemia** | LeukoStrat CDx FLT3 Mutation Assay ™ | Invivoscribe Technologies, Inc. | *FLT3* | Internal tandem duplications, *FLT3* p.D835X, p.I836X | PCR | gDNA |
| **B-cell chronic lymphocytic leukemia** | Vysis CLL FISH Probe Kit ™ | Abbott Molecular, Inc. | *LSI* probes | / | Gene fluorescent probe (FISH) | / |
| **Aggressive systemic mastocytosis** | KIT D816V Mutation Detection by PCR for Gleevec Eligibility in Aggressive Systemic Mastocytosis (ASM) ™ | ARUP Laboratories, Inc. | *KIT* | *KIT* p.D816V | qPCR | gDNA |
| **Myelodysplastic syndrome/myeloproliferative disease** | PDGFRB FISH for Gleevec Eligibility in Myelodysplastic Syndrome / Myeloproliferative Disease (MDS/MPD) ™ | ARUP Laboratories, Inc. | *PDGFRB* | *PDGFRB* gene rearrangement | Gene fluorescent probe (FISH) | / |
| **Non-transfusion-dependent thalassemia** | FerriScan ™ | Resonance Health Analysis Services Pty Ltd. | Fe concentration detection | / | MRI scan | / |

| | | | | | | |
|---|---|---|---|---|---|---|
| **Gastrointestinal stromal tumors** | Dako c-KIT pharmDx™ | Dako North America, Inc. | c-kit antibody | / | IHC antibody | / |
| **Gastic and gastroesophogeal cancer** | PD-L1 IHC 22C3 pharmDx™ | Dako North America, Inc. | PD-L1 antibody | / | IHC antibody | / |
| | HercepTest™ | Dako Denmark A/S | HER-2 antibody | / | IHC antibody | / |
| | HER2 FISH pharmDx Kit™ | Dako Denmark A/S | *HER-2/cen17* probes | / | Gene fluorescent probe mix (FISH) | / |
| **Urothelial cancer** | therascreen FGFR RGQ RT -PCR Kit | QIAGEN Manchester | *FGFR* | exon 7 (p.R248C (c.742C > T), p.S249C (c.746C > G)), exon 10 (p.G370C (c.1108G > T) and p.Y373C (c.1118A > G)) and two fusions (FGFR3-TACC3v1 and FGFR3-TACC3v3) | qPCR | / |
| **Urothelial carcinoa and triple ne** | VENTANA PD -L1 (SP142) Assay | Ventana Medical Systems, Inc. | *PD-L1* | / | IHC antibody | / |

PCR - polymerase chain reaction. gDNA - genomic DNA. FISH - fluorescence *in situ* hybridization. IHC - immunohistochemistry. CISH - chromogenic *in situ* hybridization. ctDNA - circular tumor DNA. NGS - next generation sequencing. qPCR - quantitative polymerase chain reaction. cfDNA - circulating cell-free DNA. MRI – magnetic resonance imaging.

**Supplementary Table 2.** Breast cancer associated genes (Information collected from bioinformatic databases (Clinicaltrials.gov, https://www.ncbi.nlm.nih.gov/gtr, www.fda.gov) and literature indexed in PubMed database).

| Locus | Genes | Function |
|---|---|---|
| 17q12 | *ERBB2 (HER2)* | Epidermal growth factor (EGF) receptor, family of receptor tyrosine kinases. Regulates outgrowth and stabilization of peripheral microtubules. |
| 17p13.1 | *TP53* | Code for protein p53. Protein acts as a tumor suppressor, it down regulates cell division to minimize error rate. |
| 16q22.1 | *CDH1* | Code for E-cadherin, whose function is to adheres cells. |
| 16p12.2 | *PALB2* | Important role in homologous recombination repair (HRR), it recruits BRCA2 and RAD51 to DNA breaks. |
| 11q22.3 | *ATM* | Located in the cell nucleus, it helps to control the rate of cell growth and division. |
| 22q12.1 | *CHEK2* | This protein acts as a tumor suppressor; it down regulates cell division and growth for better division control. |
| 17q12 | *RAD51D* | Involved in the homologous recombination repair (HRR) of DNA breaks during DNA replication or induced by damaging agents. |
| 2q35 | *BARD1* | It binds BRCA1 *in vivo* and *in vitro*; complex may be involved in BRCA1 tumor suppression. |
| 15q26.1 | *BLM* | Code for RecQ helicases, enzymes that attach to DNA and unwind the strands. |
| 9p21.3 | *CDKN2A* | Codes several proteins. The p16 (INK4A) and the p14 (ARF) proteins acts as a tumor suppressors. |
| 14q21.2 | *FANCM* | This gene encodes the protein for complementation of group M FANC nuclear complex group indirectly involved in cytogenetic instability, hypersensitivity to DNA cross-linking agents, increased chromosomal breakage, and defective DNA repair. |
| 11q21 | *MRE11A* | Code for nuclear protein involved in homologous recombination (3' to 5' exonuclease and endonuclease activity), telomere length maintenance, and DNA double-strand break repair. |
| 5q31.1 | *RAD50* | This protein forms a complex with MRE11 and NBS1. Complex binds to DNA; it has numerous enzymatic activities, required for non-homologous joining of DNA ends. |
| 5q22.2 | *APC* | Protein acts as a tumor suppressor, it down regulates cell division to reduce error rate. |
| 17q21.32 | *HOXB13* | The HOXB13 protein acts as a tumor suppressor. |
| 3p13 | *MITF* | This protein is a transcription factor. The protein attaches to specific areas of DNA and helps control the activity of particular genes. |

**Supplementary Table 3.** Colorectal cancer associated genes (Information collected from bioinformatic databases (Clinicaltrials.gov, https://www.ncbi.nlm.nih.gov/gtr, www.fda.gov) and literature indexed in PubMed database).

| Locus | Gene | Function |
|---|---|---|
| 12p12.1 | *KRAS* | The protein relays signals, instruct the cell to proliferate or to differentiate. |
| 1p13.2 | *NRAS* | Similar as *KRAS*, *NRAS* signals instruct the cell to proliferate or differentiate. |
| 7p11.2 | *EGFR* | EGFR receptor signaling promotes cell growth, proliferation and cell survival. |
| 5q22.2 | *APC* | Code for APC protein, which plays a critical role in several cellular processes. The APC protein acts as a tumor suppressor. |
| 3p22.2 | *MLH1* | MLH1 protein plays an essential role in DNA repair. This protein helps fix errors that are made during DNA duplication. |
| 2p21-p16.3 | *MSH2* | The MSH2 protein joins with one of two other proteins, MSH6 or MSH3. |
| 2p16.3 | *MSH6* | DNA repair protein. This protein helps fix errors that are made during DNA replication. |
| 12q24.33 | *POLE* | Involved in DNA repair and chromosomal DNA replication. |
| 3p24.1 | *TGFBR2* | TGF-β receptor complex trigger various responses by the cell, including proliferation, maturation, differentiation, cell movement and apoptosis. |
| 14q24.3 | *MLH3* | MLH genes are involved in maintenance of genomic integrity during DNA replication and after meiotic recombination. |
| 19q13.33 | *POLD1* | DNA polymerase delta plays a critical role in DNA replication and repair. |
| 1p34.1 | *MUTYH* | MYH glycosylase is involved in the repair of DNA. |
| 17q24.1 | *AXIN2* | Axin2 is inhibitor of the Wnt signaling pathway. |
| 18q21.32 | *ALPK2* | Kinase that recognizes phosphorylation sites in which the surrounding peptides have an alpha-helical conformation. |
| 16q22.1 | *CDH1* | This protein is found within the membrane that surrounds epithelial cells. E-cadherin also acts as a tumor suppressor protein. |
| 6q22.33 | *LAMA2* | Laminins help regulate cell growth, motility, and adhesion. |
| 5q14.1 | *MSH3* | MutS beta initiates mismatch repair. |
| 10q26.11 | *NOS1* | RNA-binding family member 2, it may act as a post-transcriptional repressor. |

| | | |
|---|---|---|
| 20q13.13 | *PREX1* | It has been shown to bind to and activate RAC1 by exchanging bound GDP for free GTP. |
| 15q26.1 | *BLM* | Helicases are enzymes that bind to DNA and unwind the double helix of the DNA molecule. |
| 16p12.2 | *PALB2* | This gene encodes a protein that may function in tumor suppression. |
| 3q13.33 | *POLQ* | DNA polymerase that promotes microhomology- mediated end-joining, response to double-strand breaks in DNA. |
| 9q22.32 | *PTCH1* | PTCH1 acts as a tumor suppressor. |
| 12q24.12 | *SH2B3* | SH2B adaptor family of proteins is involved in a range of signaling activities. |
| 3p21.1 | *BAP1* | BAP1 protein acts as a tumor suppressor. It functions as a deubiquitinase, which means it removes a molecule called ubiquitin from proteins. |
| 4q21.23 | *HELQ* | DNA repair protein that repairs cross-links. It also contributes to double-strand break (DSB) resistance. |
| 3p14.1 | *LRIG1* | Feedback negative regulator of signaling by receptor tyrosine kinases through increased ubiquination. |
| 22q12.1 | *CHEK2 (CHK2)* | The CHK2 protein is activated when DNA becomes damaged or when DNA strands break. Protein helps keep cells with mutated or damaged DNA from dividing, which helps prevent the development of tumors. |
| 9q34.11 | *ENG* | Code for endoglin. Localization on the surface of cells of the lining of developing arteries. |

**Supplementary Table 4.** Hepatocellular carcinoma (Information collected from bioinformatic databases (Clinicaltrials.gov, https://www.ncbi.nlm.nih.gov/gtr, www.fda.gov) and literature indexed in PubMed database).

| Locus | Gene | Function |
|---|---|---|
| 17q25.3 | *mSEPT9* | Septin family gene is involved in cytokinesis and cell cycle control; candidate for the ovarian tumor suppressor gene. |
| 5q13.2 | *CCNB1* | Regulatory protein involved in mitosis, complexes with p34 (cdc2) to form the maturation-promoting factor (MPF). |
| 10q23.33 | *CEP55* | Plays a role in mitotic exit and cytokinesis. |
| 11q24.2 | *CHEK1* | Phosphorylated by ATM in response to ionizing, ultraviolet irradiation and DNA replication. |
| 7q36.1 | *EZH2* | EZH2 enzyme forms a complex called the polycomb repressive complex-2; by turning off particular genes, this complex is involved in differentiation. |
| 17q24.2 | *KPNA2* | Adapter protein for nuclear receptor KPNB1, nuclear protein import function. |
| 6p12.1 | *LRRC1* | *LRRC1* contains code for a protein (Leucine Rich Repeat Containing 1). |
| 8p21.1 | *PBK* | Code for a serine/threonine protein kinase. |
| 2p25.1 | *RRM2* | Provides the precursors necessary for DNA synthesis, inhibits Wnt signaling. |
| 4q28.3 | *SLC7A11* | Sodium-independent amino acid transport. |
| 1q24.3 | *SUCO* | Regulates type I collagen synthesis in osteoblasts during their postnatal maturation. |
| 10q21.1 | *ZWINT* | Protein is required for kinetochore formation and spindle checkpoint activity. |
| 4q35.1 | *ACSL1* | Isozymes of this family play a key role in lipid biosynthesis and fatty acid degradation. Down regulated. |
| 1q31.2 | *CDC37L1* | Protein is located primarily in the nucleus and is likely involved in RNA transcription. Down regulated. |

**Supplementary Table 5.** Non-small cell lung cancer associated genes (Information collected from bioinformatic databases (Clinicaltrials.gov, https://www.ncbi.nlm.nih.gov/gtr, www.fda.gov) and literature indexed in PubMed database).

| Locus | Gene | Function |
|---|---|---|
| 7p11.2 | *EGFR* | Activation of receptor triggers signals that promote cell proliferation and cell survival. |
| 9p24.1 | *PD-L1* | PD1/PDL1 interaction is hypothesized to be a mechanism for immune system evasion. |
| 2p23.2-p23.1 | *ALK* | ALK receptor tyrosine kinase is thought to act early in development to regulate the proliferation of nerve cells. |
| 7q34 | *BRAF* | Protein plays a role in regulating the MAP kinase/ERK signaling pathway, involved in cell division, differentiation and secretion. |
| 17q12 | *ERBB2* | Protein is involved in the transcription process with RNA Pol I, enhances protein synthesis and cell growth. |
| 2q34 | *ERBB4* | Tyrosine- protein kinase- cell receptor regulates development of specific cell lines, gene transcription, cell proliferation, differentiation, migration and apoptosis. |
| 8p11.23 | *FGFR1* | The protein is fibroblast growth factor receptor (FGFR); Tyrosine- protein kinase plays an essential role in the regulation of embryonic development, cell proliferation, differentiation and migration. |
| 10q26.13 | *FGFR2* | An essential role in the regulation of cell proliferation, differentiation, migration, apoptosis, regulation of embryonic development. |
| 4p16.3 | *FGFR3* | An essential role in the regulation of cell proliferation, differentiation and apoptosis. |
| 7q31 | *MET* | Receptor tyrosine kinase. Regulates many physiological processes including proliferation, scattering, morphogenesis and survival. |
| 1q23.3 | *DDR2* | Receptor tyrosine kinase for ligand: fibrillar collagen. Regulates cell differentiation, remodeling of the extracellular matrix, cell migration and cell proliferation. |
| 12p12.1 | *KRAS* | Has an important role in the regulation of cell proliferation. Promotes oncogenic events by inducing transcriptional silencing of tumor suppressor genes. |
| 3q26.32 | *PIK3CA* | This gene has been found to be oncogenic and has been implicated in cervical cancers. |
| 14q32.33 | *AKT1* | AKT1 also referred to as protein kinase B, is a known oncogene. |
| 10q23.31 | *PTEN* | Controlling the tempo of the process of newborn neurons integration during adult neurogenesis, including correct neuron positioning, dendritic development and synapse formation. Potent tumor suppressor. |
| 1p13.2 | *NRAS* | Oncogene encoding a membrane protein that transports between the Golgi apparatus and the plasma membrane. |
| 15q22.31 | *MAP2K1* | Protein kinase which acts as an essential component of the MAP kinase signal transduction pathway. |

| | | |
|---|---|---|
| 19p13.3 | *STK11* | Tumor suppressor serine/threonine- protein kinase that controls the activity of AMPK family members. |
| 9q34.3 | *NOTCH1* | The signaling pathway is involved in processes related to cell fate specification, differentiation, proliferation, and survival. |
| 3p22.1 | *CTNNB1* | Protein part of Wnt signaling pathway. |
| 18q21.2 | *SMAD4* | The SMAD4 protein is a transcription factor and tumor suppressor. |
| 4q31.3 | *FBXW7* | Mutations in this gene are detected in ovarian and breast cancer cell lines, implicating the gene's potential role in the pathogenesis of human cancers. |
| 17p13.1 | *TP53* | TP53 mutations are universal across cancer types. The loss of a tumor suppressor is most often through large deleterious events, such as frame-shift mutations, or premature stop codons. |

**Supplementary Table 6.** Pancreatic cancer associated genes (Information collected from bioinformatic databases (Clinicaltrials.gov, https://www.ncbi.nlm.nih.gov/gtr, www.fda.gov) and literature indexed in PubMed database).

| Locus | Gene | Function |
|---|---|---|
| 14q11.2 | *JUB (AJUBA)* | Role in the assembly of numerous protein complexes; involved in cell fate determination, cytoskeletal organization, repression of gene transcription, mitosis, cell-cell adhesion, cell differentiation, proliferation and migration. |
| 10q24.31 | *ERLIN1* | Regulates cellular cholesterol homeostasis through the SREBP signaling pathway. |
| 12q14.3 | *HMGA2* | Transcriptional regulator involved in cell cycle regulation through CCNA2, meiotic chromosome condensation, postnatal myogenesis, involved in satellite cell activation. |
| 8q12.1 | *FAM110B* | Tumor progression suspected gene. |
| 7p11.2 | *EGFR* | Transmembrane glycoprotein- protein kinase. Epidermal growth factor receptor. |
| 3q21.3 | *MCM2* | Protein with helicase activity, involved in S phase, cell division, terminally differentiated hair cells development and cell apoptosis. |
| 3p21.31 | *TCTA* | Involved in leukemia diseases. |
| 14q13 | *SSTR1* | Somatostatin receptor couples with G proteins to inhibit adenylyl cyclase. It can stimulate phosphotyrosine phosphatase and Na(+)/H(+) exchanger. |
| 20q11.21 | *BCL2L1* | Regulate cell death by blocking the voltage-dependent anion channel. |
| 20q11.22 | *E2F1* | Transcription factor that mediate cell proliferation and p53-dependent apoptosis. |
| 7p22.1 | *RAC1* | Protein is involved in control of cell growth, cytoskeletal reorganization, and protein kinases activation. |
| 2q32.2 | *STAT1* | Transcription factor that activates the expression of the target genes, inducing a cellular antiviral state. |
